# Why understanding food choice is crucial to transform food systems for human and planetary health

**DOI:** 10.1136/bmjgh-2022-010876

**Published:** 2023-05-03

**Authors:** Krystal K Rampalli, Christine E Blake, Edward A Frongillo, Joseph Montoya

**Affiliations:** 1 Department of Health Promotion, Education, and Behavior, University of South Carolina, Columbia, SC, USA; 2 Department of Environmental Health Sciences, University of South Carolina, Columbia, SC, USA

**Keywords:** nutrition, public health, health education and promotion, environmental health

## Abstract

What, how and why people eat has long been understood to be important for human health, but until recently, has not been recognised as an essential facet of climate change and its effects on planetary health. The global climate change and diet-related health crises occurring are connected to food systems, food environments and consumer food choices. Calls to transform food systems for human and planetary health highlight the importance of understanding individual food choice. Understanding what, how and why people eat the way they do is crucial to successful food systems transformations that achieve both human and planetary health goals. Little is known about how food choice relates to climate. To clarify potential paths for action, we propose that individual food choice relates to climate change through three key mechanisms. First, the sum of individual food choices influences the supply and demand of foods produced and sold in the marketplace. Second, individual food decisions affect type and quantity of food waste at the retail and household level. Third, individual food choices serve as a symbolic expression of concern for human and planetary health, which can individually and collectively stimulate social movements and behaviour change. To meet the dietary needs of the 2050 global population projection of 10 billion, food systems must transform. Understanding what, how and why people eat the way they do, as well as the mechanisms by which these choices affect climate change, is essential for designing actions conducive to the protection of both human and planetary health.

**Summary box**

- The food systems sector is one of the major contributors to and is impacted by global climate change, one of the most profound challenges that humanity faces.
- Population-level shifts in lifestyles alongside modernisation of food environments have steered consumers towards unhealthy and unsustainable dietary patterns and ways of eating worldwide.
- Most efforts to transform food systems thus far have focused primarily on supply-side technological innovations to address climate change, with inadequate attention to what, how and why individual food choices shape food system functioning and ultimately, climate change.
- Individual food choices impact climate change in positive and negative ways through three important mechanisms: contributing to aggregate food demand, generating food waste at consumer and retailer level and catalysing collective action for social movements related to human and planetary health.
- While supply-side approaches are essential for food system transformation, there is an urgent need to understand the demand-side, specifically what, how and why individual food choices contribute to climate change and ways in which those insights can potentially be applied to meaningful and sustainable actions for climate change mitigation/adaptation.

## Introduction

Climate change poses a significant challenge for planetary and human health worldwide, and transformation of food systems will be an essential part of the solution. Planetary health broadly encompasses relationships between humanity and ecological and biophysical systems, with specific attention to the capacity of the natural environment to either support or undermine human health.^1^ From farm to table, food systems are one of the most consequential sectors impacting climate.

Food systems are the ‘sum of actors and interactions along the food value chain—from input supply and production of crops, livestock, fish and other agricultural commodities to transportation, processing, retailing, wholesaling and preparation of foods to consumption and disposal’.^2^ Food system activities (eg, producing, transporting, processing, packaging, storing of food and disposing of food waste) contribute nearly one-third (~30%) of global greenhouse gas emissions.^3^ Furthermore, food system activities have been implicated in approximately 60% of the world’s biodiversity losses and 70% of the world’s land and water use changes.^4 5^

Greenhouse gas emissions, land and water use changes and biodiversity losses have been closely linked with a multitude of environmental and atmospheric trends, collectively termed as climate change. Examples of the negative effects of climate change include increasing global temperatures, rising sea levels and changes in precipitation patterns, higher intensities and unpredictability of natural disasters (eg, floods, droughts, heat waves, tropical storms).^6^ Additionally, changes in land use (eg, deforestation, urbanisation) that disrupt wildlife habitats along with alterations in temperature and precipitation patterns bring pathogens closer to people, leading to the increased frequency and virulence of zoonotic, vectorborne and food and waterborne pathogens.^7^ Climate change does not discriminate by region or population; many around the world have and will experience the deleterious effects of extreme weather patterns, disruptions in food production and distribution and conflicts over resources. Depending on who and where, however, climate change will have differential effects on health and livelihoods, which can further widen inequities and increase hardships among vulnerable or marginalised groups (eg, women, children, minorities, etc).^8^

In the last three decades, to cater to the dietary needs of the rising global population, modern food systems have become significantly more energy-intensive and depletive of natural resources.^9 10^ As a result, global food systems are both contributors to and impacted by climate change, making food systems a key sector to target for transformation to achieve crucial planetary and human health goals. Such goals include climate change mitigation (eg, reducing and/or preventing further emissions of greenhouse gases), climate change adaptation (eg, adjusting to present and future effects of climate change) and ending malnutrition (eg, undernutrition and overnutrition).^2 8 11 12^

Historically, food systems and their related activities have not received significant attention in the public arena compared with other sectors that affect climate, such as energy (eg, heat, electricity, fossil fuels, transport) or industrial processes (eg, manufacturing of cement, chemicals, metals).^13^ Recent events such as the United Nations Food Systems Summit 2021 and the yearly United Nations Climate Change Conferences (eg, ‘COP 26’, ‘COP 27’) have brought the public’s attention to ways in which food system activities have undermined both human and planetary health.^14–16^ These events have primarily focused on agricultural practices, trade and fiscal or monetary policies that affect prices, with minimal emphasis on the role of individual food choice behaviours.^17 18^

The future of humanity depends on having a sufficient, equitable and nutritious supply of food for every person while simultaneously preserving the natural ecosystems from which food is derived. Thus, well-defined, achievable goals are necessary to transform food systems to support both human and planetary health. Even among the most well-intentioned proposals for food system transformation, there is a lack of consensus on how best to approach such a complex issue. A recurring theme observed in most food system solutions is a lack of consideration of individual food choice. Without accounting for individual food choice in food systems transformation actions, such solutions may at best, be ineffective, and at worst, have harmful unintended consequences. The purpose of this paper is to articulate the mechanisms through which individual food choice affects climate and explain why taking a consumer-driven food choice lens would enhance the ability to design and implement appropriate, effective and sustainable programmes and policies to transform food systems to benefit the health of both people and planet.

To gain a better understanding of the role of individual food choice in efforts to transform food systems for human and planetary health, in early 2022, we solicited input by conducting virtual exploratory meetings with 26 experts from a variety of organisations and institutions that work on climate change, nutrition and/or food systems. The focus of these consultative meetings was to ascertain expert perspectives on how individual food choice behaviours may be connected to climate change. These 26 experts were purposively sampled, included representatives from a variety of organisations in different sectors and countries, including low-income and middle-income countries (LMICs), and were interviewed in the context of their jobs. Participants were employed by various academic institutions, civil society organisations, private sector organisations and research institute/think tanks with global reach, which provided a diversity of perspectives. For example, some participants we spoke to were researchers on specific topics, such as animal-source food production systems, behavioural economic for behaviour change, climate change mitigation and adaptation, food marketing within food environments and regenerative agriculture. Others were spearheading initiatives to create communities of practice and advocacy activities around food systems and/or climate change in both high-income countries and LMICs. Some participants were directly involved in intervention design and community-based climate change adaptation in LMICs.

An informal interview guide consisting of fundamental questions around relationships between food choice and climate change was used. The authors explained the purpose of the consultation, elicited responses from the participant(s) and took extensive notes on the conversations that ensued. In drafting this manuscript, we drew on the themes, experiences and insights discussed during those meetings, supplemented with a comprehensive review of related literature. The information included in the manuscript does not use direct quotes from meeting attendees nor does it contain any personal identifiers. Prior to submission, a near-final manuscript draft was sent out to attendees for feedback and approval.

## Individual food choices are an important element of all food systems

Individual food choices play an integral role in the functioning of food systems. Food choice is defined as a decision-making process whereby individuals consider, acquire, prepare, distribute and consume foods and beverages.^19 20^ Decisions that individuals make about what, how and why to select specific foods are constrained by what is available and accessible within local food environments.^21^ The complexity of the food choice decision-making process increases with greater availability and accessibility to foods.^3 20 22^ Varied causal factors have been posited as drivers of food choice (eg, taste, habit, culture, cost) at different levels of influence (eg, personal, community, market) with varying explanatory value.^19 20 23 24^ It is commonly assumed that resource-poor populations or those residing in remote or rural areas do not have the ability to make any choices about what to eat. Even in the most disadvantaged settings, however, people can and will exercise agency in their food choice behaviours to the extent possible, given their real and/or perceived constraints and limitations (eg, cost, time to acquire or prepare, facilities to prepare) and personal perspectives (eg, cultural acceptability, taste preferences, health needs).^25^ Nevertheless, those who are most vulnerable to the deleterious effects of climate change are often those who have the least amount of agency in their food choices. For example, in a study conducted in rural Guinea, when faced with multiple constraints, including limited time and unpredictable incomes as unskilled day labourers, artisanal miners chose from various energy-dense, nutrient-poor, commercially produced foods, snacks and drinks that were readily available near their mining work sites. Other lower cost foods in raw form requiring more time for preparation were available, but miners sometimes prioritised time and convenience over cost and effort required to procure and prepare the food.^26^ Thus, acknowledging that food choices are never made in a vacuum is important, where only availability and accessibility drive behaviour; rather, the choices that people make about what, how and why to eat certain foods involve a complex series of considerations that are deliberate arbitrations of priorities and related tradeoffs (eg, personal vs familial preferences, health vs convenience, time vs money).^19^

The global food industry has a major influence on individual food choice through the global food system, local food environments, marketing and advertising and dietary guidance.^27^ Food industry actions shape national and transnational food and nutrition policies, which affect which foods are available and the information people receive about what and how much of certain foods to consume or avoid (eg, food-based dietary guidelines).^28 29^ In high-income countries such as the USA, food lobbyist groups (eg, Grocery Manufacturers Association, National Pork Producers Council, National Restaurant Association) routinely exert influence to shape food and nutrition policy.^30^ An example of food lobby influence is seen in the *Dietary Guidelines for Americans*, a leading source for evidence-based nutritional recommendations developed by the government (eg, United States Department of Agriculture and Department of Health and Human Services).^31^ In 2015, the guidelines were slated to include information about environmental impacts of animal-source foods and health benefits of a plant-based diet.^32^ Despite demand for such guidance from the public, industry pressure resulted in the removal of language about sustainability and environmental impacts of different dietary choices based on concerns about the quality of evidence and economic impacts.^33 34^ As a major contributor to what, how and why people eat the way they do, in recent decades, the global food industry has invested substantial resources for food science research (eg, to develop novel and appealing food products) and food marketing across various platforms (eg, television, radio, internet/social media, mobile phones, outdoor advertising) to influence individual food choices.^35–37^

Over the last century, modernisation of food systems for a growing population has occurred alongside various changes in lifestyles, livelihoods and food environments. Delivery of nutrition-sensitive and nutrition-specific interventions (eg, homestead food production programmes, school feeding programmes, large-scale food fortification programmes, salt iodization programmes) have had promising health impacts for some population groups, including reductions in severe acute malnutrition and some nutritional deficiencies.^38 39^ Other food system changes have engendered population-level shifts in food choice that have undermined preferences for foods and ways of eating congruent with both human and planetary health.^40 41^ These negative dietary changes are commonly referred to as the nutrition transition, which is characterised by an increased consumption of foods containing unhealthy levels of refined carbohydrates, sodium and saturated or trans fats and are typically heavily processed to enhance flavour, shelf life, aesthetics and palatability of foods at the expense of nutritional content.^42 43^ On a global scale, the nutrition transition results in growing congruence of unhealthy eating patterns across both high-income and LMICs.^42^ Such changes in global food consumption have implications for both human and planetary health.

The intersection of concerns about human and planetary health is increasingly focused on the global rise of red meat consumption, defined by WHO as, ‘all mammalian muscle meat, including, beef, veal, pork, lamb, mutton, horse and goat’.^44 45^ In high income countries, higher red meat consumption has been found to be associated with obesity and risk of diet-related non-communicable diseases (NCDs), including type 2 diabetes, cardiovascular disease and some cancers.^42 46 47^ Increased demand for beef, in particular, has negative climate and environmental implications due to the agricultural, industrial and transportation systems and processes needed to support the production and distribution logistics across the supply chain (eg, land and water use changes, pollution from single-use plastic packaging needs, biodiversity loss, greenhouse gas emissions).^41 48^ The implications of moderate beef consumption in LMIC or among vulnerable populations (eg, food insecure) is less clear. In some circumstances, animal husbandry may have positive effects on human and planetary health.^49 50^ Thus, refining and clarifying goals for human and planetary health with respect to beef production and consumption is warranted (box 1).

Box 1 **Beef has turned into a battleground for climate and health activists. Does it need to be this way?**

Demand for beef has been elevated in high-income countries for several decades. In low-income and middle-income countries, however, demand for beef has been increasing at unprecedented rates.^102^ Although there is evidence that diets high in beef may lead to NCDs, there is a considerable share of the world’s population that does not receive adequate amounts of essential nutrients for proper body functioning through their diets, such as heme iron, zinc and protein. These nutrients and others are easier for the body to absorb and thus more bioavailable by consuming animal-source foods, particularly beef.^47 50^ How beef is produced, however, makes a difference in climate change and human health. In many cases, relatively small-scale ranches are run as ‘cow-calf operations’, where cattle are bred and fed on the land and then sold to feedlots, areas where hundreds of cattle are aggregated together and fed corn and/or other high-energy grains.^128^ Direct environmental impacts of the feedlot system include substantial greenhouse gas emissions, water usage and deforestation to create ample land for intensive farming of cattle feed grains.^44 129^ But is beef production inherently problematic? Although some argue that beef production is within acceptable limits, and can even be beneficial to the environment, many scientists assert that beef production is a serious problem given current demand.^49 130^ Many involved in the production of beef state that cattle have the unique ability to digest cellulose-rich plant material grown on non-arable land and convert the indigestible nutrients into meat consumable by humans.^50^ Cattle raised on pastureland (grassy areas designated for grazing) return valuable nutrients to the soil, stimulate carbon sequestration and can be integral for the germination of many deep-rooted grasses.^49 131^ Compared with the beef derived from their feedlot-raised cattle counterparts, beef from pasture-raised cattle contains more fatty acids and antioxidants that are known to be beneficial for human health.^132 133^ This system of regenerative agriculture, however, is rarely used in the commercial production of red meat and in most cases, even when regenerative agricultural methods are fully or partially employed, greenhouse gas emissions from cattle methane production is still estimated to be far greater than the potential for carbon sequestration.^134^ Currently, most beef is not produced using sustainable methods.^130^ If individual food choice were to shift to an increased demand for more nutrient dense beef in smaller quantities, a shift in agricultural practices that promote rather than harm the environment are potentially viable. This shifting of consumer expectations to higher quality beef and away from large-scale convenience consumption could potentially lead to more responsible beef production, however, this is unlikely to occur without a catalyst. As a result, beef production remains a major contributor to climate change.^134^

Successful food system transformation for human and planetary health requires clear and achievable goals for individual food choice behaviours. Identifying dietary patterns and ways of eating that contribute positively to both human and planetary health is an emerging area of study. Many elements of healthy dietary patterns are consistent with recommendations for climate-friendly diets (eg, less ultra-processed foods, less red meat, more plant-based foods).^51^ In 2019, the EAT-Lancet Commission launched dietary recommendations that would allow for the projected 2050 global population of 10 billion to be fed and still satisfy various markers of planetary health.^52 53^ Some analyses of the EAT-Lancet planetary diet noted the impracticality of these recommendations for most LMICs, which have a disproportionate burden of undernutrition, poverty and food insecurity.^54^ For example, one of the major critiques of the EAT-Lancet planetary diet was the omission of affordability, which is a key driver of food choice for most of the world and presents a significant challenge to promotion of diets that will satisfy both planetary health and human health metrics.^46 54^ Further efforts are underway to provide dietary guidance that considers context-specific constraints and population-level needs to achieve both human and planetary health.^55–57^ This guidance will be invaluable for goal setting to achieve food system transformation for both human and planetary health.

What, how and why people choose to eat the way they do in the coming years will be crucial for guiding food systems transformation. Thus, it is imperative that researchers, practitioners and policymakers understand the mechanisms by which individual food choices can shape food system functioning, activities and patterns. By applying a food choice lens to actions for food systems transformation to improve both human and planetary health, we enhance our ability to account for nuances in context and behaviour that can contribute to success or failure of such actions.

## How individual food choices contribute to climate change

Understanding individual food choice is a key step in the design and implementation of actions to transform food systems to achieve climate and health goals. Individual food choice contributes to climate change in three significant ways, described below.

First, any one individual’s food choices contribute to an aggregate population-level demand for food in the global marketplace and that in turn influences what items are produced, promoted and sold. The total supply and demand for food can be viewed through a market lens, where the collective willingness and ability of sellers to produce and sell a good or service and the collective consumers’ willingness and ability to purchase a certain good or service mutually reinforce one another in either positive or negative ways.^58^ In simple terms, when consumers buy more of a certain product, sellers continue to produce and distribute more of that product (eg, digital music subscriptions such as Spotify or Apple Music), or when consumers do not buy more of a certain product, sellers respond by discontinuing production and distribution of that product (eg, cassette tapes with the advent of digital music).^59^ Sometimes demand can rise and fall at different time periods, based on cultural trends (eg, the resurgence of demand for once obsolete vinyl records).^60 61^ When faced with reduced demand for certain goods and services, producers may try to use creative and/or aggressive marketing techniques, including offering free samples, membership club benefits, coupon promotions, rebates, buy-one-get-one discounts and other incentives on different media platforms to stimulate demand.^62^ Despite these efforts, if demand does not materialise, producers may ultimately scale down or eliminate production of the good or service. For certain goods and services, a high supply or low price does not necessarily lead to a higher demand for those goods and services.^63^

The collective impacts of individual food choices deeply shape food system operations and food production decisions along various domestic and international food value chains, all of which have far-reaching implications for climate change.^64–66^ For example, water and land use and agricultural inputs (eg, fertiliser, seeds) decisions are made regularly based on market demand for certain foods and public sector investments in certain commodity crops. Such decisions subsequently impact both domestic and international trade policies and patterns.^67^ Consumer demand also affects policies relevant in food production, such as subsidies. As subsidies help to keep food prices low for the consumer, they tend to improve both accessibility and affordability. Most of the foods that are heavily subsidised, however, require highly efficient food production systems that use substantial volumes of agricultural inputs and emit large volumes of greenhouse gases.^66 68^

Second, food waste at individual, household and retail levels are significant contributors to greenhouse gas emissions.^69 70^ Food loss is commonly defined as food that is spilled, spoilt or lost in the food supply chain prior to reaching the consumer. It commonly occurs during production, postharvest, processing and distribution. Food waste, on the other hand, refers to food that is suitable for human consumption but for various reasons, is discarded at the household or retail level. Some examples of food waste include unsold food from retail stores, plate waste, uneaten prepared food, kitchen trimmings and by-products from food and beverage processing facilities.^71^ Additionally, food packaging materials (eg, cardboard, plastic, Styrofoam) used to keep food hygienic and appealing to consumers is also often discarded and comes with a sizeable environmental footprint.^72^ Approximately 14% of global food production is lost prior to reaching the retail level.^73^ On reaching retailers and consumers, about 17% of food is wasted.^70^ In high-income countries, a significant driver of food waste is misperceptions about food safety, quality and freshness, which can lead to edible food being disposed of earlier than necessary.^74^ In LMICs, much of food waste is due to lack of cold storage for perishable foods, leading to spoilage and disposal.^75^ By understanding the wants and needs of retailers, households and individuals, and how food choices are made, programmes and policies can be designed to better address the longstanding issue of food waste and its contributions to climate change.

Third, individual food choice decisions may catalyse social movements. Individuals who adopt and promote food choice behaviours to mitigate climate change influence the behaviours of others.^76^ Particularly in the high-income countries, people commonly scrutinise the lifestyles of themselves and those in their social networks as they become more conscious of various connections between their lifestyles, including their personal food choices, and planetary health.^77^ Visible changes in individual food choice, such as reducing one’s meat consumption, are often among the first steps people make towards achieving a personal health goal. This choice can also simultaneously serve as a symbolic act to display to others that one is willing to cooperate in the collective pursuit of achieving planetary health goals.^78^ Collective action can be fostered by groups of people who publicly display their individual food choice behaviours as a symbolic expression of their shared vision and concern for human and planetary health.^79^ Social and cultural movements have the highest potential for effectiveness when they can emphasise common goals and avoid devolving into the polarising us-versus-them intergroup bias and related stigmatisation, which can enable groups to come together and advocate for positive change in food systems, human health and climate change.^80^

There are several examples in public health where individual behaviours or actions have inspired larger social, cultural and eventually, political movements. For instance, the organisation Mothers Against Drunk Driving, which began in the early 1980s by two mothers whose children were killed in alcohol-related traffic accidents, has been a powerful lobbying force to pass local and national alcohol regulations in the USA. Some of their most significant policy wins in the last four decades include: raising the minimum drinking age, setting up sobriety checkpoints and influencing public opinion to support stricter alcohol regulations through awareness campaigns and partnerships with government agencies and taskforces (eg, National Highway Traffic Safety Administration, Centers for Disease Control, Presidential Commission on Drunk Driving, etc). Mothers Against Drunk Driving began as a grassroots effort that continues to influence the public sector.^81 82^ A similar path for food systems transformation is possible for nations, states, communities and social groups as they collectively come to recognise that what and how we eat has implications for human and planetary health.

From a health equity perspective, individual food choices and how those choices affect climate change cannot be ignored. Low-income consumers contribute substantially to aggregate food demand through their food choices. For example, the popularity of discount chain stores such as Walmart can largely be credited to low-income consumers who contribute to an increased demand for cheaper goods, including ultra-processed foods.^83^ Food waste patterns and causes differ based on relative wealth and affluence. For example, when people are wealthy enough to spend at least US$6.70 per day per capita, they are more likely to waste food, and the amount increases as income increases.^84^ Vulnerable populations, although they typically have the most unmet needs in food and nutrition, are less empowered and typically less likely to catalyse social movements related to food justice and climate change action.

## Competing goals create challenges for transforming food systems

Competing goals have created numerous and ongoing challenges across global food systems. Some of these competing goals include scaling the reach and volume of the world’s food supply to reduce or eradicate hunger, creating conditions to promote optimal human health (eg, reducing or preventing diet-related NCDs, improving accessibility, affordability and availability of food that meets national and international dietary guidance), assuring a safe and traceable food supply, maintaining profitability for producers, improving smallholder farmer livelihoods and preserving planetary health (eg, reducing greenhouse gas emissions and biodiversity loss, promoting sustainable food production).^85^

Until recently, most actions seeking to transform food systems for improving human and planetary have emphasised producer responsibilities. Such actions focus on the ways in which food is supplied to consumers through modifications to food production, food production methods and value chains. Food system solutions oriented towards supply entail the use of biotechnology, adaptive farming practices including breeding crop varieties resistant to natural disasters or pests, carbon sequestration and other methods that would increase efficiency of production, transportation and processing of food with the purpose of either climate change mitigation or adaptation.^86 87^ Implementation of such actions has been and continues to be extensively researched, and successful scale-up of these solutions are likely to have a direct impact on greenhouse gas emissions and other direct contributors to climate change.^88^ Producer actions in food systems designed to address climate change, however, are not free of challenges. They are resource-intensive, highly dependent on geography and existing infrastructure and often require political will and sustained cooperation at higher levels of governments.^86 87^

In the last century, much of the world has grappled with hunger and malnutrition, and addressing issues related to supply have long been considered the remedy. Throughout the 20th century, several technological and scientific breakthroughs made it possible to produce enough food to feed the world’s growing population, with the emphasis on energy intake and a few selected nutrients (eg, protein). The Green Revolution resulted in food system transformations that vastly improved food security and reduced hunger through the scale-up of production of high-yield varieties of staple grains (eg, rice, wheat).^89^

Crop intensification gave rise to industrial agriculture and food processing methods that reduced uncertainty and increased uniformity in food systems.^90^ Some industrial food processing has been beneficial in reducing food waste by improving food security and food safety, increasing shelf life of perishable foods and enabling the foods to undergo long-distance transportation.^91 92^ As food companies sought to have more control over the quality and appearance of food through processing, however, they launched marketing campaigns emphasising uniformity and cosmetic perfection of their food products. Manipulation of consumer preference and establishment of food quality standards resulted in consumers demanding foods that are predictable (eg, always taste and appear as expected).^93^ Consumer demand for foods with predictable taste and aesthetic profiles corresponded with widespread utilisation monocropping and other resource-intensive inputs necessary for industrialised agriculture (eg, synthetic fertilisers and pesticides, water) that compromise soil quality and deplete the nutritional value of food for human health and contribute to the environmental degradation that is currently observed^93 94^ (box 2).

Box 2 **Is organic better?**

Organic food is considered by many to be good for human and planetary health. The marketing and sale of organic food, originally described to consumers as ‘chemical-free’, has been occurring for over 80 years. In 1990, the United States Department of Agriculture (USDA) ‘Organic’ certification was officially established to provide consistent guidance to farmers and consumers.^135^ Yet, the question remains: Is organic food better for people and the environment? For people desiring food without pesticide residues or chemical fertiliser remnants, they will find what they are looking for in organic foods. Organic farming is likely safer for farm workers as they are not exposed to significant levels of toxic pesticides and agrochemicals that conventional farming necessitates.^136^ For example, agrochemicals that were once considered safe, such as the common weed killer glyphosate, have been linked to some cancers.^137^ Relationships to planetary health, however, are difficult to assess and the scale matters. Although the gap is shrinking, organic farms tend to produce less food per acre than conventional farms, so some researchers argue that organic farming produces more carbon emissions per pound of food than conventional farming.^138 139^ Synthetic fertilisers have been shown to make crops grow quicker compared with organic fertilisers, and pesticides commonly used in agriculture can control pests and diseases that can result in crop failure.^140^ There is some evidence suggesting that organic farm productivity is improving and may be more resilient amid natural disasters, economic downturns or political conflicts.^141–145^ Organic farming originally emphasised the importance of using natural rather than artificial processes (eg, composting instead of using synthetic chemicals for fertiliser).^135^ These practices corresponded with a regard for soil as a living organism with many components. The USDA Organic certification, however, refers to a set of practices, not philosophies. Furthermore, it does not reference many of the tenets central to chemical-free farming, such as the treatment of soil as a living entity.^146 147^ The push for organic farming has led to some unintended consequences. Due to the vague language of the USDA certification, some organic farms more closely resemble their conventional counterparts than the original model of non-chemical farms.^148^ There has been a proliferation of other forms of organic certification created by private, third-party certifiers.^149^ For some farmers, the price of obtaining certification can be costly, so organic farming may still be practised without the official certification.^150^ Usually, the increased costs associated with organic food production and certification are absorbed by consumers, making organic food less accessible to lower-income individuals. Lastly, in recent years, there has been significant controversy generated by governments mandating organic requirements, some of which have incited widespread protesting, riots and job losses.^151 152^ Overall, the health and environmental benefits of organic production and consumption are difficult to measure, leaving consumers to make their food choices based on observable characteristics, personal values, certifications and economic realities.

During the time when the scale-up of industrial agriculture and food processing was starting, concerns developed worldwide about food safety, minimisation of losses, cutting costs in value chains and support for farmer livelihoods.^95^ The implementation of minimum standards for food quality and hygiene helped alleviate some consumer doubts about the safety of the food supply. For example, food quality and hygiene standards enabled a surge in single-use plastics for packaging to transport, store and serve food and beverage products, which has now been implicated in widespread environmental pollution.^72 96^ Furthermore, as local food production systems were displaced by the emphasis on economies of scale and production efficiency, agriculture subsidy programmes were implemented to protect farmers by absorbing some of the risks or uncertainties that are inherent with farming.^68^ By ensuring that food always looked, smelled and tasted the same, consumers could trust that the food they were ingesting was safe, neglecting to account for the food not being particularly nutritious. The downside of uniformity in food production value chains was that potential unintended but negative health and planetary consequences, such as loss of biodiversity and increases in processed products containing unhealthy ingredients (eg, added sugars, sodium, saturated fats, artificial colours, preservatives and flavours), were overlooked by stakeholders looking to maintain trust among consumers.^93^

## Why take a food choice perspective to food systems transformation?

Consumer-driven actions are essential to transforming food systems to meet the dual goals of improving both planetary and human health, but they are not the only piece of the puzzle.^87 88^ An integrated strategy incorporating actions from both the suppliers and the consumers is needed, with the recognition and appreciation of where, how and why individual food choice fits into the larger picture. Considering that food system activities contribute to approximately one-third of the world’s greenhouse gas emissions, the status quo is unsustainable in the long-term for preservation of human health, livelihoods and overall planetary health. There is consensus that the current food system, dominant food production methods and food consumption patterns are extractive and depletive of the earth’s resources. There is also recognition that food systems need to transform to become both climate-smart and nutrition-sensitive to ensure there is enough nutritious food produced in sustainable ways to feed everyone on the planet. Creating a climate-smart and nutrition-sensitive global food system, however, would require mitigation and adaptation actions that consider the ways that climate change affects nutrition and subsequently, human health.^8^ As we seek to reform food systems, we should aim to achieve complementary, not competing goals for both human and planetary health (eg, reduced incidence of NCDs, nutritionally adequate diets, affordable and accessible foods, low greenhouse gas emissions, reforestation, increased biodiversity), with a keen awareness that actions must be tailored, depending on the country, context and community.^97^ An individual’s food choices are often an expression of personal beliefs, values, culture, emotions and identity, and are driven by availability and accessibility, as well as considerations of cost, taste and convenience.^20 24 98^ Producer-focused efforts that fail to take individual food choice decision-making processes into account or end up being misaligned with consumer demand can yield disappointing results^99 100^ (box 3).

Box 3 **Establishing food markets in food deserts without consideration of drivers of food acquisition behaviour leads to intervention failure**

It is commonly believed that increasing local access to supermarkets in US food deserts would lead to healthier food choices among low-income communities who have limited access to healthy food where they live. Between 2004 and 2016, various policies provided funds to build over 1000 supermarkets across 35 states in neighbourhoods classified as food deserts with the goal of improving the quality of dietary intake through purchase of fresh fruits and vegetables and other healthy foods.^99^ The desired effects were not achieved. Low-income families still chose unhealthy foods and bypassed the new local supermarket to shop farther away at their preferred venues, typically supercentres or clubs (eg, Walmart, Sam’s Club), where they could purchase shelf-stable food (usually processed, less healthy items) in bulk for lower prices.^153 154^ The policies did not account for how and why people make food choice decisions. In practice, individuals optimised their limited resources by shopping at venues with the widest variety of foods they preferred or were familiar at the best prices. They also considered presence of other goods and services (eg, clothing, banking, pharmacy) to minimise the number of times they had to travel. These features were more influential drivers of food acquisition than proximity to residence.^153 155^ After withdrawal of initial investment, many of these local supermarkets closed due to lack of customers. While the installation of supermarkets in food deserts was a well-intentioned effort to transform the local food systems through direct investment on the supply end, understanding of how and why individuals in the target communities made food purchasing decisions was lacking. This led to a significant wastage of resources and ultimately did not improve utilisation of healthy food options in food deserts.

Various demand-side actions have been proposed to reduce the impact of climate change and improve human health. Some examples include public campaigns by health agencies in high-income countries (eg, the USA, Western Europe) to promote plant-based diets and reduce red meat consumption, emphasising health reasons. The rationale of targeting high-income countries is based on data that there is significantly higher per capita red meat consumption which is linked to the hefty NCD burdens and measurable elevations in greenhouse gas emissions and deforestation.^101 102^ Such campaigns alone have been largely insufficient to persuade individuals to eat less red meat.^103 104^ Similarly, although the average high-income country consumer is aware of and is moderately concerned about climate change affecting their life, prior environmental and behavioural research has demonstrated that the same average consumer does not consistently act on those concerns in a tangible way, a gap between attitudes and behaviour.^105 106^

Other actions designed to persuade consumers to make more sustainable purchasing decisions include alterations in government feeding programmes (eg, ‘Meatless Monday’ school feeding programmes), flooding the market with plant-based meat alternatives (eg, Impossible Burger, Beyond Meat) or other protein sources, or food-related fiscal policies (eg, excise taxes that increase the cost of meat products).^88 107–110^ Addressing demand for red meat and environmental concerns by incorporating knowledge about food choice decision-making processes would provide insight to inform actions likely to yield successful changes in food choice behaviour but will require innovative thinking. Local food movements have been cited as a promising attempt to promote radical transformation of food culture through grassroots approaches. One example of innovative food choice behaviour change with implications for human and planetary health comes from the vegan soul food movement in the Southern USA^111 112^ (box 4).

Box 4 **The vegan soul food movement to preserve health, tradition and identity**

Traditional soul food is an extremely important part of identity, tradition and culture for much of the Black American population in the Southern USA. The cuisine is also famous for being extremely high in salt, fat and meat, which is detrimental for both human health and planetary health. Recently, Black American chefs and entrepreneurs began the vegan soul food movement as a grassroots effort to address the disproportionately high rate of obesity, diet-related non-communicable diseases and generally poor health outcomes in their respective communities without losing critical aspects of their culture rooted in the cuisine. So far, the movement has shown promising results for health in intervention studies and is gaining momentum in various cities around the Southern USA.^111 112^ This is an example of a grassroots movement to change individual food choices achieving meaningful population-level changes in diet with implications for both human and planetary health.

Other examples of tapping into food movements that emphasise culture, social identity and shared history to create positive changes with implications for human and planetary health:

- *Finland*: https://www.theatlantic.com/health/archive/2015/04/finlands-radical-heart-health-transformation/389766/.
- *USA (Native Americans)*: https://www.indigikitchen.com/.
- *West Africa (Fulani people)*: https://fulanitestkitchen.com/.
- *Pacific Island Food Revolution (South Pacific)*: https://www.pacificislandfoodrevolution.com/.

An important lesson to glean from local food movements is that there are ways to guide consumers towards healthier food choices and ways of eating that do not undermine their own health and the health of the planet. Some of these food choices and ways of eating would entail examining our history and cultural practices around food to learn about what and why our ancestors ate the way they did, whether it was to meet their basic human needs, to embrace a specific type of identity or culture, to feel good or for another reason. Having such information may help consumers embrace food that is of higher quality and culturally acceptability, going beyond the cheapest or trendiest items in their local supermarkets. Thus, food system actions oriented towards increasing or reducing demand for certain foods should be compatible with the population and food environment context in which the action(s) would be delivered. For example, interventions in a context where undernutrition is prevalent and significant subsets of the population require the type and volume of protein that can only be derived from animal-source foods to meet daily nutritional requirements will look different from interventions in a context where overnutrition and NCDs are prevalent and animal-source foods are regularly overconsumed.^50^

Viewing food systems transformation for human and planetary health through a food choice lens is valuable when considering people’s food acquisition, storage and consumption practices and how those contribute to food waste generation. While there are several types of food losses that occur throughout the value chain from farm to table, there is significant wastage (~50%) at the consumer stage during acquisition, storage and consumption.^113^ Studies in LMICs and high-income countries found that perspectives on food safety and food quality significantly influence food choice behaviours, particularly around what to buy, where to buy, which vendors to buy from and how long to store food before disposal.^114 115^ From a climate change perspective, misplaced food safety and quality concerns have been leading contributors to food waste at the consumer level.^74^ One of the significant food safety and quality concerns that has led to unnecessary food waste is the perception that visually imperfect food is unsafe to consume.^74 116^ While those concerns may be valid in some instances, they are not always, and some initiatives have sought to understand and changes those misconceptions (box 5).

Box 5 **The ‘Love Food, Hate Waste!’ campaign used a personalised strategy, working with individuals to help them examine what, how and why they were wasting so much food**

In the mid-2000s, there was a heightened awareness by the UK public that food waste at the consumer level was a serious issue for health and climate. To that end, the Waste and Resources Action Programme (WRAP), the UK’s chief agency for waste and resource efficiency management, underwent research and later, implemented the ‘Love Food, Hate Waste!’ campaign in 2007. First, they sought to understand the factors that went into what, how and why food in UK households was being wasted. WRAP found that it was important to raise awareness of food waste as a major contributor to climate change, as well as highlighting the benefits of saving money, and creating messages that cater to people’s personal convictions and values to act on environmental issues. They also found that many people believed they lacked the knowledge or skills to manage the food in their houses, which is why so much went to waste, so part of the intervention involved engaging with consumers on a one-to-one basis, helping them learn and gain the skills to plan their own meals and make grocery lists to prevent frivolous spending and subsequent waste. The campaign proved to be successful at reducing waste behaviours by individuals and households.^156 157^ By working with individuals and households and understanding their food acquisition, preparation, storage and consumption behaviours, WRAP and the ‘Love Food, Hate Waste!’ campaign was able to work with consumers to meet them where they were at to help them understand why reducing food waste was important for their life and the planet.

A food choice perspective could be used to create and disseminate various call-to-action messages that could help mobilise communities, challenge entrenched ideologies, promote climate and food justice activism and work on locally led adaptation measures.^117 118^ Considering that climate change events have differential effects on population groups and geographic regions worldwide, successful adaptation and mitigation strategies must be locally led with communities who are on the frontlines of climate change.^119^ For example, in Fiji, in 2014, due to rising sea levels and subsequent flooding, a village relocation project moved 132 coastal residents 2 km inland. This decision was not easy, as villagers were concerned about how their traditional lifestyles, including diets, culture and livelihoods which were rooted in activities related to aquaculture (eg, obtaining, selling, sharing, cooking and consuming fish) would be affected by this internal displacement that would also lead to limited ocean access.^120^ Those in charge of the relocation project listened to the villagers and their concerns and collaborated directly with them as well as national and international organisations to cocreate acceptable solutions to this community that had positive livelihood and lifestyle effects, assuring them that while some things might change, other aspects of their traditional lives would remain intact even with the relocation. Some tangible solutions that came out of this village relocation project that were implemented included inland fish farming ponds, new farming methods using different salt-resistant crop varieties and provision of copra dryers for commercial opportunities for this community.^121^ Having the food choice perspective was helpful in this scenario for cocreation of acceptable solutions for a community whose identities and livelihoods were heavily dependent on naturally occurring phenomena that was undergoing significant stress.

Finally, using a food choice perspective for food systems transformation actions to improve human and planetary health can be useful to help forecast future unhealthy food demand patterns to thwart unintended negative consequences. For example, population-level food choices in Nepal were modified significantly in a brief period, with higher demand for ultra-processed foods, such as packaged snacks and instant noodles. Many of these foods were provided as part of food assistance programmes in the aftermath of the 2015 earthquake when people were highly food insecure and trying to survive. After prolonged exposure during a crisis, however, taste preferences evolved, particularly among children, to favour those foods which are now ubiquitous in many Nepalese food environments. The food assistance intervention, while well-intentioned, disrupted existing dietary patterns and introduced new food choices that now pose challenges to the public’s health.^122^ By accounting for how and why individual food choice behaviours are occurring and the types of preferences that may arise in a natural disaster or conflict, future food assistance programmes may consider that a similar phenomenon as what has happened in Nepal may arise elsewhere, and tailor their programmes to promote food assistance that is less disruptive to food systems while still aligned with planetary and human health goals.

Achieving complementary health and planetary goals requires a team effort on the global scale, with participation from members across various sectors, governments and communities. Individuals are the main end-users of the food system, and individual food choices form the foundation of total consumer demand and should be understood to develop strategies to reduce food waste and stimulate social and cultural movements for positive change. Consequently, the choices of individuals should be considered as a focal point of food system transformation.

## Conclusion

In accounting for food choice behaviours and their drivers in the formulation of actions to transform food systems to better serve human health and reduce the deleterious effects of climate change, several tangible steps must be taken. Evidence should be gathered and synthesised as to what, how and why people eat the way they do in various settings, countries, contexts and among different population groups and at different times to inform food system transformation efforts. Understanding how individual food choice influences aggregate food supply and demand can improve efforts to monitor current trends and forecast future trends relevant to climate change and food systems, such as supply chain disruptions. Furthermore, actions to mitigate or adapt to climate change on the demand-side require careful planning and targeting of interventions (eg, tailoring of relevant and impactful behaviour change communication campaigns). Considering that individual food choice has been implicated in how and why there is significant food wastage at the individual, household and retailer levels, creating opportunities for ways to assist consumers in using their food more efficiently at the acquisition, preparation, storage and consumption stages is important in efforts to mitigate climate change and improve human health. Tangibly, this would involve understanding and measuring food choice behaviours to determine what, how and why specific foods are wasted to develop improved preservation methods (eg, enhanced packaging or storage containers, improvement in expiration date forecasting, improvements in processing methods such as canning, drying or freezing) and provide guidance for messages to help alleviate fears about food safety among consumers by informing them how to better preserve their leftovers, use their foods more efficiently and understand the meaning of the labels to reduce the waste they generate. Lastly, understanding the extent of awareness and engagement local populations have with food-related health and climate concerns can intensify efforts towards policymakers to act at the macro-level on matters of significant relevance, such as food systems transformation and climate change mitigation and adaptation.

In the policy arena, current debates on food systems and climate change appear to frame planetary health through a climate change lens. This simplified framing is done in isolation of other major issues affecting, or affected by food systems, such as various economic or trade policies, wars and other political upheavals (eg, Ukraine-Russia conflict), pandemics (eg, COVID-19) and other macro-level issues on the national, regional or global stages. For example, most proposed climate change actions at the national or international level involve targeting carbon dioxide emissions, despite credible evidence that methane gas emissions from livestock, paddy rice cultivation and other agricultural activities are significantly more damaging to the atmosphere because methane is denser and more potent in global warming than carbon dioxide.^123^ Some of the more popular diet-related solutions to climate change also may come with unintended consequences. For instance, plant-based meat alternatives are heavily processed with various additives and other chemicals designed to mimic the taste and texture of actual meat. Despite being plant-based, these foods are still high in energy, saturated fat and sodium.^124^ Although the efforts to increase demand for plant-based meat alternatives through promotion of the environmental benefits compared to their animal-source food counterparts, there is limited evidence to confirm such claims.^125^ When consumed in significant quantities, plant-based meat alternatives, much like animal-source foods, can still lead to negative human health outcomes, including obesity and diet-related NCDs.^126 127^

Most actions related to the ongoing climate and health crises that have aimed to transform food systems have been directed towards producers. Such actions often involve the use of novel technological innovations or implementation of policies to address inefficiencies in food value chains that contribute to climate change. Although continuing to strengthen responsibilities of producers to transform food systems is important, focusing exclusively on producers and neglecting the influence of individual food choice behaviours would be to the detriment of the success of food systems transformation proposals, including the promotion of diets that are both healthy and sustainable. Both system-level changes (eg, producer-focused actions, technological innovations) occurring in tandem with individual-level changes (eg, consumer-focused actions) would be ideal for impactful food systems transformation. By understanding the decision-making process undergirding food choice, avenues to mitigate climate change through the three mechanisms can be explored.

The world’s population is projected to reach nearly 10 billion by the year 2050.^52^ To meet the dietary demands of this enormous number of people, food systems will need to transform to protect both human and planetary health. Understanding individual food choice behaviours as critical components of aggregate food demand, determinants of food waste and as important symbolic expressions for grassroots advocacy is essential. In our increasingly complicated, globalised and heavily interdependent world, changes in individual food choice behaviours are central to sustainable food systems transformation.

## Data Availability

All data relevant to the study are included in the article.
